# Standardization of the assessment process within telerehabilitation in chronic diseases: a scoping meta-review

**DOI:** 10.1186/s12913-022-08370-y

**Published:** 2022-08-02

**Authors:** Blandine Chapel, François Alexandre, Nelly Heraud, Roxana Ologeanu-Taddei, Anne-Sophie Cases, François Bughin, Maurice Hayot

**Affiliations:** 1 University of Montpellier, Montpellier Research of Management, Montpellier, France; 2 Direction de La Recherche Clinique Et de L’Innovation en Santé, Korian ; GCS CIPS, 800 Avenue Joseph Vallot, Lodève, France; 3 Toulouse Business School, Toulouse, France; 4 PhyMedExp, University of Montpellier, INSERM, CNRS, CHRU Montpellier, Montpellier, France

**Keywords:** Telerehabilitation, Health technology assessment, Evaluation, Chronic disease, Scoping meta-review

## Abstract

**Background:**

Telerehabilitation (TR) interventions are receiving increasing attention. They have been evaluated in various scientific areas through systematic reviews. However, there is a lack of data on how to standardize assessment and report on their domains to guide researchers across studies and bring together the best evidence to assess TR for chronic diseases.

**Aims and objectives:**

The aim of this study was to identify domains of assessment in TR and to qualitatively and quantitatively analyze how and when they are examined to gain an overview of assessment in chronic disease.

**Methods:**

A scoping meta-review was carried out on 9 databases and gray literature from 2009 to 2019. The keyword search strategy was based on *"telerehabilitation*", *“evaluation"*, *“chronic disease"* and their synonyms. All articles were subjected to qualitative analysis using the Health Technology Assessment (HTA) Core Model prior to further analysis and narrative synthesis.

**Results:**

Among the 7412 identified articles, 80 studies met the inclusion criteria and addressed at least one of the noncommunicable diseases (NCD) categories of cardiovascular disease (cardiovascular accidents), cancer, chronic respiratory disease, diabetes, and obesity. Regarding the domains of assessment, the most frequently occurring were “*social aspect”* (*n* = 63, 79%) (e.g., effects on behavioral changes) and “*clinical efficacy”* (*n* = 53, 66%), and the least frequently occurring was *“safety aspects”* (*n* = 2, 3%). We also identified the phases of TR in which the assessment was conducted and found that it most commonly occurred in the *pilot study* and *randomized trial* phases and least commonly occurred in the *design*, *pretest*, and *post-implementation* phases.

**Conclusions:**

Through the HTA model, this scoping meta-review highlighted 10 assessment domains which have not been studied with the same degree of interest in the recent literature. We showed that each of these assessment domains could appear at different phases of TR development and proposed a new cross-disciplinary and comprehensive method for assessing TR interventions. Future studies will benefit from approaches that leverage the best evidence regarding the assessment of TR, and it will be interesting to extend this assessment framework to other chronic diseases.

**Supplementary Information:**

The online version contains supplementary material available at 10.1186/s12913-022-08370-y.

## Background

At a time when life expectancy is increasing, chronic or noncommunicable diseases (NCDs) are also on the rise [1]. The latest World Health Organization (WHO, 2015) report indicates that NCDs, like mainly heart and lung disease, obesity, cancer, and diabetes, are responsible for 16 million premature deaths (before the age of 70) each year. As the leading cause of morbidity, disability and mortality in industrialized countries, NCDs constitute a real public health problem, and their prevention, treatment and risk factors have become major international issues [2]. Along with other well-established treatments such as pharmacotherapy, supplemental oxygen, or noninvasive ventilation, nondrug interventions, such as rehabilitation, have become the standard of care for these diseases. *Rehabilitation* is a comprehensive intervention focused on the patient's needs [3]. There are two major objectives in rehabilitation. The first objective is to restore or optimize functional ability for a chronic patient. This goal is achieved by the implementation of exercise training to improve the symptomatology of the disease. The second objective is to encourage health-promoting behaviors. For this purpose, therapeutic patient education, which supports self-management of the disease, is used in several activities like smoking cessation, nutrition and physical activity [3].

At present, due to the use of information and communication technologies (ICT), the use of telemedicine and the potential effectiveness of mobile applications [4, 5], *telerehabilitation* (TR) is increasingly attracting the attention of policymakers, payers, healthcare professionals, patients and the scientific community. TR is a subcategory of telehealth that uses different types of communication modalities, which can be divided into synchronous such as live video, and asynchronous, such as e-mail or remote monitoring [6], to provide clinical rehabilitation services from a distance [7]. TR is emerging as an innovative approach to providing remote care and to deploying rehabilitation [8–12], to improve accessibility and continuity of care and to educate patients about adherence and long-term maintenance of the beneficial effects of rehabilitation [13, 14]. TR is an intervention that emerged at the end of the 1990s [15], and it represents a powerful tool for improving the management of daily practice and the creation of networks between health structures such as hospitals or clinics, and services.

However, despite undeniable evidence of the contribution of TR, many TR interventions still fail and rarely achieve technology adoption [16, 17], user engagement and intervention effectiveness [18–20]. Considering the several elements that could explain this failure, it is clear that TR suffers from a lack of exhaustive development in its many dimensions, and that a gap may exist between the design of TR and the moment when it will be used [21]. Indeed, assessing TR is a complex matter for several reasons: TR is a broad concept that requires multidisciplinary collaborations with many stakeholders [22], who must simultaneously consider the dimensions that characterize TR intervention: technological, clinical and others like ethical, cost-effectiveness, social [23–26]. In addition, health information systems should be assessed with the same rigor as a new drug or treatment program to prevent decisions about future deployments of ICT in the health sector from being determined by social, economic, and/or political circumstances rather than by robust scientific evidence [27]. Thus, we must be able to evaluate TR interventions while they are being designed, developed and deployed [27]. Hence, in assessing TR, an extensive appraisal including these different dimensions is needed in each phase of the technology’s life cycle [27–29], as was reported in the e-health evaluation model by Enam et al., (2018) [30].

The aim of this scoping meta-review [31] was to systematically map recent research to contribute to the standardization of the assessment process within TR applications. First, to identify the domains of TR assessment for chronic diseases, we used a comprehensive evaluation framework with a multidisciplinary approach called the Health Technology Assessment (HTA) Core Model [32]. The HTA includes ten domains and appears to be the most complete framework. In addition, we identified the phases of TR interventions in which assessment occurs by mapping out the content of the reviews and grouping the phases of intervention with similar objectives, activities, or results. On this basis, our two research questions were as follows:

- 1. What domains of evaluation have been identified in the literature on TR for chronic disease?
- 2. What are the assessment domains of the different phases of TR?
  Finally, the findings allow us to present a novel way of examining the assessment of TR interventions and could provide a reference and information for policymakers, clinicians and researchers regarding the development of assessment guidelines.

## Methods

This study is based on a new method, the scoping meta-review [31]. This method combines the scoping review and meta-review methods. *Scoping reviews* entail reviewing the emerging literature to provide an initial indication of the size and nature of the available literature on a particular topic [33, 34]. *Meta-reviews* involve synthesizing evidence from a set of systematic reviews [35, 36]. We first considered performing a simple scoping review, considering that the field is diverse and complex [37]. However, during the initial extraction of the data, we noticed the existence of numerous systematic reviews on various aspects of TR assessment. Thus, it seemed relevant and feasible to undertake a scoping review of the systematic reviews related to TR assessment approaches [31]. The advantage of relying on systematic reviews is that they can provide a solid and reliable synthesis of work in the field [37].

### Protocol

We followed the guidelines of Levac et al. (2010), updated from the initial work of Arksey and O'Malley (2005). In addition, we wrote the protocol using the “Preferred Reporting Items for Systematic reviews and Meta-Analyses Extension for Scoping Reviews” (PRISMA-ScR) [38]. Levac et al. (2010) suggest that the protocol should not be designed as a rigid tool and strictly applied because it is likely that as familiarity with the literature is increased, researchers will be able to redefine search terms and undertake more sensitive literature searches. The process is not linear but iterative, requiring us to engage with each stage in a reflexive way and, where necessary, to repeat the steps to ensure that the literature is covered comprehensively. Hence, in our study, the protocol was used as a guide, and we followed it when necessary [39]. The final protocol is not publicly available; however, it can be made available upon request to the corresponding author.

### Eligibility criteria

The methodological approach of the scoping review allowed for the identification and alteration of the inclusion and exclusion criteria as articles were selected. Inclusion and exclusion were performed first on the basis of article selection through the "title" and "abstract" filters and then by reading the articles in full.

Studies that were in line with the *inclusion criteria* (1) were in the systematic literature review format; (2) addressed at least one of the NCD categories of cardiovascular disease (cardiovascular accidents), cancer, chronic respiratory disease, diabetes, or obesity identified on the basis of the prevalence and importance of the common behavioral risk factors such as smoking, poor diet, sedentary lifestyle, and harmful use of alcohol (according to the figures provided by the WHO); (3) addressed TR in the sense of the definition given in the rationale (see the introduction); (4) included features of the definition of rehabilitation presented in the rationale; (5) contained at least one intervention offering physical activity as part of a multidisciplinary approach; (6) dated from 2009 to 2019 because the field of TR must take into account the increasing evolution of ICT in health such as connected objects and mobile devices; (7) reported in French or English (criteria adopted for practical reasons: material from other languages was excluded due to the cost and time involved in translation); and (8) used an adult population 18 + years old.

Studies that were in line with the *exclusion criteria* (1) presented interventions without technology or limited to a telephone follow-up approach; (2) presented a single study or were opinion papers, draft syntheses, abstract/conference proceedings (oral presentations and posters), chapters, discussions, letters, books available electronically, and theses; (3) were those where it was impossible to identify the type of intervention performed; (4) addressed an intervention with technology limited to the physical activity dimension alone (without multidisciplinary approach) or did not contain a physical activity dimension; or (5) addressed methods/requirements without real evaluation related to TR or for which it was impossible to locate the full text.

### Information sources

The field of TR must take into account the growing ICT evolution in health such as connected objects and mobile devices; thus, we included studies published only between January 2009 and October 2019. We conducted extensive literature searches in the electronic bibliographic databases most likely to contain the type of study we are looking for. The databases are multidisciplinary, covering fields from computer science to health science: MEDLINE (PubMed), Web of Science, Cochrane Library, ABI, Business Source Premier, PsycINFO, Science Direct, Academic Search Premier, and SPORTDiscus. We conducted additional searches in the gray literature (a) by consulting the reference lists of the included studies and (b) by searching repositories of gray literature: CADTH, Directory of Open Access Journals (DOAJ), Occupational Therapy Systematic Evaluation of Evidence (Otseeker), International Prospective Register of Systematic Reviews (PROSPERO), OpenSIGLE (OpenGrey), and the New York Academy of Medicine Library’s Grey Literature Report. The research strategy was planned and carried out through structured team discussions and in consultation with a university librarian so that the strategy could be refined in light of the initial results. We exported the final search results to Zotero (5.0.95.1, Roy Rosenzweig Center for History and New Media, Fairfax, Virginia), a bibliographic database. This software facilitated the management of the research, particularly the identification and removal of duplicates.

### Search—identification of relevant studies

We identified the keywords through, on the one hand, the medical subject headings (MesH) providing the controlled vocabulary for MEDLINE/PubMed and, on the other hand, other keywords, which we call free vocabulary. Free vocabulary was added based on the expertise of the different team members but also by reading the abstracts in first intention (the first reading performed on the abstracts) if it seemed relevant and necessary. The search strategy was based on the terms "telerehabilitation" AND "evaluation" AND "chronic disease" and all their synonyms (see Additional file 1). Each database was searched individually. The keyword search strategy, based on the use of the Boolean operators AND / OR as well as ti(title) and ab(abstract), is described below.

### Selection of sources of evidence

To increase consistency among the 5 reviewers, pairs were created to independently evaluate article titles and abstracts for inclusion in the study. Evaluators met at the beginning, midpoint and end of the process to discuss issues and uncertainties related to the selection of potentially relevant studies and to re-evaluate and refine the research strategy if necessary. We then independently reviewed the full papers for inclusion. Disagreements over study selection and data extraction were resolved by consensus and discussion with other reviewers to make a final decision on inclusion if necessary.

### Data charting process

To begin, the first author developed a data table. We used a conceptual framework to guide the data extraction (Additional file 2). Subsequently, the other coauthors discussed and validated this table to determine the variables to be extracted. Once this first version was finalized, three members of the evaluation team tested the table by independently collecting data on three articles to share their perspectives concerning the dimensions to be collected. Next, two of these reviewers conducted data collection on 10% of the corpus of selected articles and discussed the results, continuously updating the data charting table in an iterative process. We then carried out a second calibration exercise testing the % agreement, with a predetermined level of agreement (70% to 80%) [38]. The concordance determined by the Kendall concordance coefficient (W) in SPSS software was greater than 80% (mean: 87%; Kendall's W = 0.8697). The first researcher thus finalized the coding alone, and any disagreements and questions were resolved through discussion between the two reviewers. Moreover, by charting the content of the reviews and grouping those with similar objectives, activities, and/or outcomes, thematic analysis could be used to determine whether the reviews focused on certain phases of the technology life cycle rather than others.

### Data items

The data extracted from the articles are as follows: author(s), year of publication, location of study, design of review (narrative review, descriptive review, scoping review, meta-analysis, systematic review, theorical review, etc.), title, type of pathology, fields of technology (m-health, e-health, etc.), definition of technology, tools associated with technology (SMS, apps, web, etc.), end-users, number of studies included (study design and participants), whether type of evaluation allows for consideration of and focus on phases of the technology life cycle (design, pretest, pilot study, randomized trial, post-implementation), key findings, and critical appraisal of researcher (if applicable). For each review, we extracted the presence and number of evaluation domains based on the HTA model (see the following paragraph). In accordance with the PRISMA-ScR, we did not perform a quality assessment or quality evaluation, as this is not essential for scoping review methodologies. Thus, the methodological rigor of the published articles was not a criterion for inclusion or exclusion for two reasons. First, scales measuring this quality, such as Assessing the Methodological Quality of Systematic Reviews (AMSTAR), tend to focus on experimental studies (interventions). Given the other possible aspects of TR (e.g., ethical or economical), we did not want to discriminate against those studies. Second, these scales are relevant if we need to compare the effectiveness of the interventions studies. This is not the aim of our study; rather, we were interested in the various aspects of the TR assessment beyond medical effectiveness.

### Critical appraisal of individual sources of evidence: the hta (healthcare technology assessment) core model framework [32]

We needed an analytical framework, a multidisciplinary approach that could include all domains of TR assessment, which encompasses medical as well as non-medical domains, to know what was being evaluated and to break down the silos of the assessment dimensions. This early assessment can be important because it can influence the TR project before the implementation. For this purpose, we chose the Health Technology Assessment (HTA) Core Model [32] from among the evaluation frameworks available in the literature [40–44] because it suggests what kinds of information one can find in an HTA report, and its definition encompasses the dimensions of multidisciplinarity and comprehensiveness. The definition of a *health technology assessment* is as follows: *“a multidisciplinary process that summarises information about the medical, social, economic and ethical issues related to the use of a health technology in a systematic, transparent, unbiased, robust manner” (p13)* [32]. The structure of the information collected is as follows: the domains of assessment (the broad framework within which the technology is considered), the topics of assessment (more specific considerations in one of the domains), and the issues raised (even more specific considerations on one of the topics that may be similar to research questions in scientific studies). The structure of the model is based on the combination of these three points to define the different assessment elements and facilitate a shared understanding of what belongs to HTA. Additional file 2 below provides an overview of the ten domains described in the HTA core model: health problem and current use of technology, description and technical characteristics of technology, safety, accuracy, clinical effectiveness, costs and economic evaluation, ethical analysis, organizational aspects, social aspects, and legal aspects.

### Methods of handling and summarizing the data

A qualitative synthesis of the included studies is conducted to chart the literature on the domains of TR assessment. The data are summarized using descriptive tables of the categories developed from the HTA framework. Additionally, a qualitative inductive and content analysis approach allowed us to bring out other elements of TR assessment (completing the existing framework). Finally, thematic analysis was applied by mapping out the content of the papers and grouping the phases of intervention with similar objectives, activities, or results. The objective of the analysis was to understand whether the researchers emphasize some phases over others during evaluation and, if so, what phases are most frequently evaluated during an intervention.

## Results

### Selection of sources of evidence

**Fig. 1:**
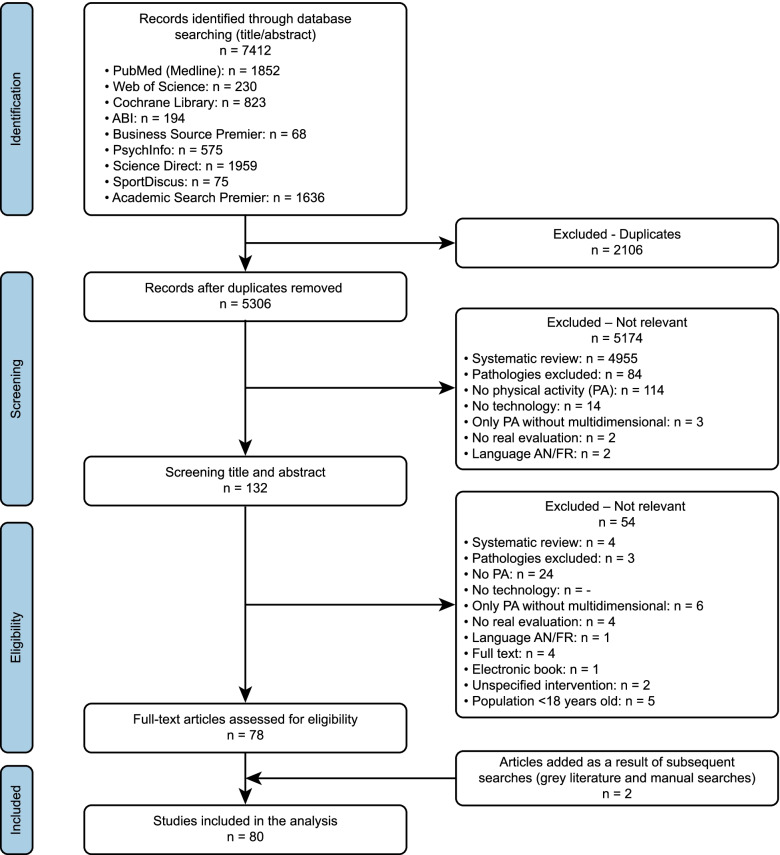
PRISMA flow chart for the study

The search of the 9 databases generated 7412 results (Fig. 1). After elimination of duplicates, 5306 publications remained. The review of the titles and abstracts led to the exclusion of 5174 publications, leaving 132 publications requiring screening on the basis of the full text. Full text screening helped to remove an additional 54 publications, leaving 78 articles. The most common reason for excluding citations during full-text screening was that the studies did not include physical activity in their interventions (*n* = 24). Subsequently, 2 articles were added from the gray literature and manual research. A total of 80 publications remained (Fig. 1), all of which focused on one or more domains of TR assessment and required further analysis.

### Characteristics of the reviews included and reports according to the eunethta template

*Year of publication and geographical distribution—*Table 1 shows the number of reviews included by year bracket between 2009 and October 2019. Few reviews were published between 2009 and 2013 (*n* = 14, 18%). Most of the articles were published from 2014 onwards, and more than one-third of the articles (*n* = 26, 33%) were published from 2018—2019, the last two years studied. The majority of the reviews (*n* = 31, 39%) were from Europe, followed by North America (*n* = 25, 31%), Oceania (Australia and New Zealand) (*n* = 13, 16%), Asia (mainly China) (*n* = 9, 11%) and South America (*n* = 2, 3%).

**Table 1:** Characteristics of the included documents

Characteristics	No. of reviews (*n* = 80)	Percentage (%)
**Year of publication**		
2009—2011	7	9
2012—2013	7	9
2014—2015	19	24
2016—2017	21	26
2018—2019	26	33
**Location of study**		
Europe (including UK)	31	39
North America	25	31
Oceania	13	16
Asia	9	11
South America	2	3
**Pathologies concerned**		
Diabetes	45	56
Cardiovascular diseases	38	48
Chronic respiratory diseases	25	31
Cancers	20	25
Obesity	19	24
**Design of the review**		
QSR	52	65
QSR and MA	14	18
Umbrella (meta-review)	4	5
Scoping review	4	5
MA	3	4
QSR and meta-synthesis (meta-ethnography)	1	1
Realist review	1	1
Descriptive	1	1
**Field of technology (as stated by the authors)**		
m-health	33	41
e-health	16	20
Telehealth	16	20
Web (internet)-based intervention	6	8
m-health and e-health	3	4
Telemedicine	2	3
Telerehabilitation	2	3
Digital health intervention	2	3

*MA* Meta-analysis, *QSR* Qualitative systematic review

*Pathologies concerned—*The main categories of NCDs were cardiovascular diseases (heart attacks), cancers, chronic respiratory diseases (such as chronic obstructive pulmonary disease or asthma), obesity and diabetes. Fifty-four articles (68%) focused on a single pathology, and 26 (32%) focused on >  = 2 pathologies. More than half of the articles dealt with diabetes (*n* = 45, 56%), followed by cardiovascular disease (*n* = 38, 48%). One-third of the reviews were on chronic respiratory diseases (*n* = 25, 31%). A quarter (*n* = 20, 25%) of the reviews addressed cancer, closely followed by obesity (*n* = 19, 24%) (see Table 1).

*Types of systematic literature reviews—*As shown in Table 1, more than 80% of the reviews were additionally derived from qualitative systematic reviews (*n* = 52, 65%), meta-analyses (*n* = 3, 4%) or the performance of both at the same time (*n* = 14, 18%). For the other reviews, we found scoping reviews (*n* = 4, 5%), meta-reviews (umbrella) (*n* = 4, 5%), a meta-ethnography and a descriptive review.

### Areas of intervention and definitions

In our research, the term "telerehabilitation", according to the definition given in the rationale, was found under different names in each of the included reviews. The most-used terms were rather generic: "m-health" (*n* = 33, 41%), "e-health" (*n* = 16, 20%), and "telehealth" (*n* = 16, 20%) or, more rarely, "web-based intervention/rehabilitation" (*n* = 6, 8%), "e-health/m-health" (*n* = 3, 4%), "digital health intervention" (*n* = 2, 3%), and "telemedicine" (*n* = 2, 3%). The term "telerehabilitation" appeared only twice (3%). Systematic reviews used different definitions corresponding to the field of intervention stated in the research. Despite the sometimes disparate definitions, the numerous reviews nevertheless provided an evaluation of different studies (with different interventions) that may correspond to the definition of TR given in the rationale. In this way, some authors raised the issue of the difficulty of determining the element of effectiveness of the TR interventions evaluated [45].

### Types of associated technological tools

We classified the technological tools mobilized into several categories (see Additional file 3). In most studies, a combination of several tools was identified to enable the interventions to be carried out [41, 46–48]. Mobile and internet/website applications were most commonly used, with 51 articles (17% for each). Short message system tools followed, with 47 articles (14%). Often, in addition to these first three tools, phone calls (*n* = 39, 11%), digital devices (e.g., connected objects) (*n* = 36, 11%), and emails (*n* = 29, 9%) were added. Less frequently, it was also possible to identify the following tools: videos/images (*n* = 22, 6%), videoconferences (*n* = 14, 4%), and social networks (*n* = 14, 4%). More rarely, we found that studies used personal health reports (*n* = 4, 1%) or other technological tools, such as logbooks, virtual reality, or digital libraries.

### Completeness of the reports according to the EUnetHTA template

Given the number of sources included in the scoping meta-review, the relevant data from each source are provided in Additional File 4. On average, we found a total of 3 HTA domains evaluated per review. Table 2 shows the number of HTA domains appearing in the reviews. Briefly, the most represented domains were *social aspect*, in 79% (*n* = *63)* of the reviews, and *clinical efficacy*, in 66% (*n* = 53). *Ethical analysis* and *safety aspects* were both evaluated only in 3% (*n* = 2) of the reviews studied, and *accuracy* was not represented in our data.

**Table 2:** EUnetHTA HTA Core Model Domains included in the reviews

Domains evaluated	No. of domain appearances	Percentage (%)
HTA Core Model domains		
1. Social	63	79
2. Clinical effectiveness	53	66
3. Health problem	46	58
4. Description and technical characteristics of technology	37	46
5. Cost and economic evaluation	14	18
6. Organizational	12	15
7. Legal	12	15
8. Ethical	2	3
9. Safety	2	3
10. Accuracy	-	-

### Telerehabilitation assessment domains identified

In this section, we present the TR domains that review authors evaluated. To understand these domains, we mapped them using the principles derived from the EUnetHTA HTA framework (see Methodology). After extracting the domains during the qualitative analysis, we classified them into nine categories: *social aspects, health problem and current use of technology, description and technical characteristics of technology, costs and economic evaluation, organizational aspects, legal aspects, ethical analysis,* and *safety.* Table 3 shows these domains and the key aspects of their measurement.

**Table 3:** Description of identified telerehabilitation assessment domains

Domain assessed	Key subjects of measurement
Social aspects	*Major life areas:* Technologies are seen as opportunities to identify similar user communities and share experiences among peers [49]. Effect on psychological distress [50], stress management, fatigue [50, 51], knowledge of treatment and chronic disease [52–55]. Encouraging but varied effects on self-efficacy [53, 56, 57]. Effects on behavioral changes in PA, diet, medication adherence, and smoking [53, 57–62]. *Individual:* Ten reviews examine the acceptability of the intervention [63, 64] mainly to the patient as an end user [58, 65, 66], and 15 reviews study satisfaction [58, 67, 68]. Facilitators and individual barriers are also studied [45, 69]. *Communication:* Evaluation of the usability of technologies during the development process [25, 46, 58, 63, 70]. Need for targeted technology [65] and stimulation of user engagement, motivation and involvement over time [68, 71, 72], and the quality of patient-caregiver interaction [64, 65, 68, 73]
Clinical effectiveness	*Health outcomes:* Benefits and unanticipated negative effects of telerehabilitation interventions compared to standard interventions (usual care). Outcomes include postintervention mortality [74, 75], clinical results (blood lipids, blood pressure, hemoglobin A1c, weight and BMI) [20, 47, 53, 58, 76], quality of life [51, 56, 66, 68], anxiety, depression [51, 58, 75, 77–80], and physical functions (exercise capacity, exercise tolerance); presented in the short [47, 54, 81], medium and/or long term [82, 83]. *Patient satisfaction:* Willingness to reuse or recommend the technology [51, 55]. *Comparative accuracy of a replacement technology:* More specific or safer technological intervention than an older or comparable technological intervention (with more features, feedback, educational messages, or combinations of technological tools) [20, 65, 81]
Health problems and current use of technology	*Target condition:* Differences in the effectiveness of the intervention according to the various targeted pathologies, possible differences from one pathology to another [50, 65]. Example of type 1 diabetes and type 2 diabetes [20, 76]. *Utilization:* differences in use between countries or a lack of education in low-income countries [66, 84]. Identification of the applicability and acceptability of telerehabilitation in primary care, general practice and hospital settings [85]. *Other:* Only one study focused on evaluating the actors involved in the design of the technology (i.e., a team of IT developers) [86]
Description and technical characteristics of technology	*Features of the technology:* General information (name, type of device, language, etc.) [48, 67, 86], purpose of using the technology (e.g., to promote behavioral change) [45, 58, 79, 87], technical characteristics (ergonomics, functionalities, interoperability) [88–91]. *Investments and tools required to use the technology:* Type of operating system and its availability [49, 71, 84, 86, 92]; the brand and relevance of technological tools [23, 25, 59]. *Training and information needed to utilize the technology:* Protocols, educational materials, recommendations, and documents developed to make the intervention appropriate for the target population [48, 65, 67, 70, 92–94]. Need to establish feasibility, accessibility and usability studies [89]
Costs and economic evaluation	*Unit costs:* Related to the unit costs of the resources used (e.g., technology acquisition costs or the cost of specific actions) [49, 65, 70, 84, 86, 95, 96]. *Outcomes:* Health cost outcomes by type of telerehabilitation intervention [97], compared to a control group [68], to prevent, predict or minimize exacerbation [98]. *Cost-effectiveness:* Intervention that can be cost-effective under certain conditions [56, 95]. Despite being minimally studied, "urgent" need to performed controlled and homogeneous trials [99]
Organizational aspects	*Process:* Monitor care outcome processes, such as maintenance of the behavioral effects of the intervention [65], clinic attendance, the effectiveness of the chronic disease surveillance system or the compliance of tools used to improve clinic attendance (e.g., SMS reminders) [95]. Interest in having a multidisciplinary team trained in motivational feedback [100]; fund technology-oriented studies and encourage proposals from interdisciplinary groups of researchers [65]. *Structure:* The effects of the implementation of interventions on hospital admissions, the use of health resources [72, 101, 102], clinical workload and workflow, and dependence on technology for work [103]. *Management:* Interest in proposing multiple models of patient management (e.g., integrating alternative models) based on evidence, responding to the needs and profile of patients [68, 77]. Take into account the intentions of future caregivers to integrate technological tools into their practice [65, 89]
Legal aspects	*End-user*: Identify the various target populations [53, 69, 77, 104] and those that are poorly studied [48, 53] to carefully examine the possibility of generalizing new modalities of intervention and their potential dissemination. *Privacy of the patient and authorization and safety:* Describe procedures to ensure the security and storage of private data [64] and identify whether problems occur in the private sphere [67]. *Legal regulation of novel/experimental techniques:* Adapt the development of new mobile applications to regulations (e.g., medical devices) [89]. *Regulation of the market:* Identify whether reimbursement of intervention systems is possible and by whom [65]
Ethical analysis	*Principal questions about the ethical aspects of technology:* Spreading the use of technologies (e.g., ethical challenges of privacy and data security)[25]
Safety	*Technology-dependent safety risks:* Identify potential problems with the reliability and validity of information entered into the technology by the patient or caregiver; identify the number of adverse effects of interventions in patients [63]. *Use- or user-dependent safety risks:* Identify potential complications that may arise due to certain functionalities (e.g., misinterpretation of information sent) [65]

### Additional non-hta TR domain

The HTA framework was not developed specifically for TR, and during the inductive and thematic analysis and by comparing results between reviewers, we found that 95% of the reviews (*n* = 76/80) evaluated the *"interventional aspect"*. This interventional aspect combines the characteristics of the interventions or their functionalities and the application of recommendations and theoretical foundations to construct these interventions. Therefore, we decided to add this assessment domain to complement the HTA framework.

### Characteristics of the intervention

The main characteristics of the intervention can be classified according to the strategies used. An average of 5 strategies was identified per review: educational information (*n* = 61; 76%), communication with others (*n* = 53; 66%), self-management (physical activity, diet, medication adherence, smoking) (*n* = 64; 80%), feedback and self-monitoring (*n* = 48; 60%), use of prompts/cues (reminders and alerts) (*n* = 38; 48%), exercise training (*n* = 32; 40%), psychosocial support (*n* = 12; 15%), stress management (*n* = 10; 13%), patient assessment (*n* = 8; 10%) and others. Additional file 5 gives a sample of the intervention characteristics identified in ten reviews.

### Application of recommendations and theoretical foundations for behavior change

Clinical recommendations suggest that ongoing behavioral support is necessary for lifestyle changes to be sustainable [58]. Many reviews (*n* = 31; 39%) present intervention characteristics based on specific theories/conceptual frameworks for designing and optimizing TR interventions [53, 84, 94]. The behavior change theory (BCT) developed by Abraham & Michie (2008) is the most widely used behavior change theory in technological applications (*n* = 13/31) [41, 60]. The most used BCTs include “goal setting”, “self-monitoring of behavior”, “information about health consequences”, “social support”, and “feedback and monitoring”. The transtheoretical model [105] and social cognitive theory [106] are the next most applied (*n* = 10/31). Many other theories are mentioned more sporadically, such as self-efficacy theory [58], the theory of planned behavior—reasoned action [94], social ecological theory [47], social support theory [53], the self-management model [72], self-determination theory [84], and cognitive behavior theory [64].

### Phases of the telerehabilitation assessment process

**Fig. 2:**
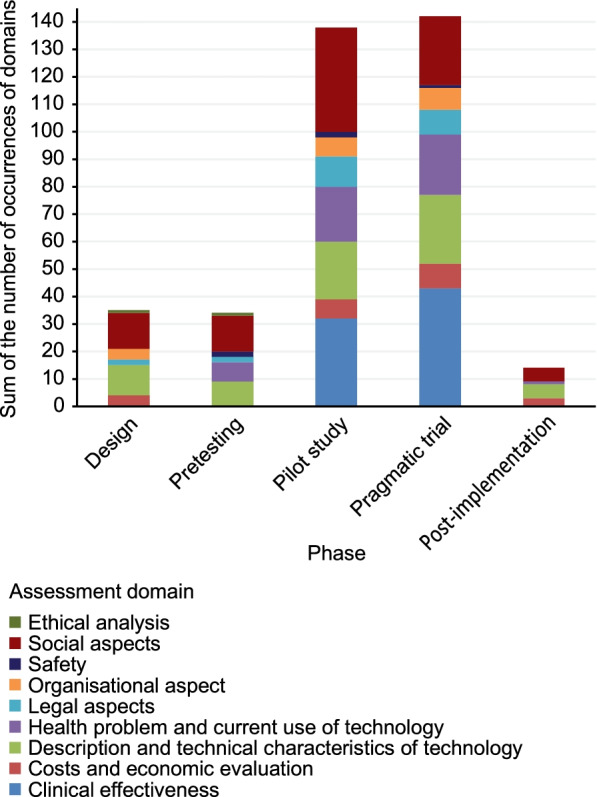
Telerehabilitation assessment process

To answer our second research question, we focused on how the assessment was conducted in the distinct development phases of TR: *design, pretest, pilot study, randomized trial* and *post-implementation*. This led us to develop the *telerehabilitation assessment process* (Fig. 2), which illustrates the accumulation of evidence by crossing the assessment domains with the distinct development phases of TR. The domains of assessment (i.e., *health problem and current use of technology, description and technical characteristics of technology, safety, clinical effectiveness, costs and economic evaluation, ethical analysis, organizational aspects, social aspects,* and *legal aspects)* vary in each phase. The results show that assessment is mainly carried out in the *pilot study* and *randomized trial* phases. For example, during the *pilot study* phase, the focus of assessment shifts primarily to the *social aspect,* followed mainly by *clinical effectiveness.* On the other hand, assessment is rarely carried out in the *design, pretest*, and *post-implementation* phases. When a TR intervention initiates with the *design* phase, the decisions are made based on the evaluation of the *description and technical characteristics of technology, social aspects, costs and economic evaluation, organizational aspects*, *legal aspects* and *ethical analysis.* The *health problem and current use of technology* and *safety* domains appear in the *pretest* phase. Although reviews of the post-implementation phase are limited, this comprehensive evaluation process can be used to gradually accumulate evidence that could be used to make future decisions.

## Discussion

This scoping meta-review was conducted to identify the different domains of TR assessment for chronic diseases and provide a comprehensive view of TR assessment through the analytic framework of HTA. The results indicate that many systematic reviews are generally focused on a limited number of assessment dimensions.

In identifying and summarizing the main domains of assessment, we highlighted the multidisciplinarity and comprehensiveness of the assessment of TR. Our study shows that nine out of the ten domains composing the HTA framework have been explored by TR reviews (*social aspects, clinical effectiveness, description and technical characteristics of technology, health problem and current use of technology, costs and economic evaluation, legal aspects, organizational aspects, safety,* and *ethical analysis*). This result reflects the relevance of this framework for our specific analysis.

Much of the focus centered on the domains of *social aspect* and *clinical effectiveness.* Together, they represent 48% of occurrences, though they constitute only 20% of the HTA domains (*n* = 2/9 domains). This reflects an imbalance in the assessment of the different domains. To date, research in these two domains has relied primarily on randomized controlled trials (RCTs) to assess TR. The performance of an RCT is considered the “gold standard” in research [107], and the RCT is a unique approach of achieving lifestyle changes in patients with chronic diseases [65]. Despite this major interest, some researchers have questioned their usefulness given the complexity of assessing TR interventions [107]. Our results also show that the least frequently occurring domains were *safety* and *ethical analysis.* Although our review highlights some promising emerging results that may help commissioners, developers, and users manage risks and improve patient safety [63, 65], several studies have shown that mobile medical applications (apps) could compromise patient safety [108, 109]. Future research could develop a risk framework that users, developers, and other stakeholders can use to assess the likely risks posed by specific apps in a specific context [110]. Finally, concerning *ethical analysis,* despite the fast-paced growth of TR, only a few articles propose suggestions to practitioners for addressing ethical challenges such as acquiring compliant software, receiving training, creating informed consent procedures, and using an ethical decision-making model [111].

Regarding our choice to mobilize the HTA Core Model, we evaluated its relevance and operationality with respect to TR. This framework had not yet been applied in the field of TR, so we sometimes had difficulty classifying some of the data using the HTA domains. Indeed, there was ambiguity regarding some items that could be classified under multiple domains at the same time. For example, in this framework, the description of the *social aspects* domain includes the effects on behavioral changes in physical activity and diet. These elements could also be classified under *clinical effectiveness* for a health or rehabilitation expert. We can also take a critical view of the results with respect to the frequency of the appearance of certain domains (e.g., the *social aspects* domain is present at a greater frequency than the *clinical effectiveness* domain). Furthermore, with regard to the model, it would be interesting to consider its supplementation or comparison with other validated frameworks. For example, the literature proposes many (more or less comprehensive) approaches to e-health [40], m-health and even telemedicine [38, 105–107] assessment to assist decision makers who want to introduce and use this technology.

Moreover, we identified an additional non-HTA domain, the *interventional aspect*, which defined several attributes of TR intervention assessment. The majority of published reviews (*n* = 76, 95%) examine the different characteristics of interventions that engage the patient and foster the success of TR to promote behavior change and positive health outcomes. A number of reviews provide encouraging evidence about BCTs and their benefits for the improvement of physical activity outcomes [60]. In contrast to this literature, a recent meta-review highlighted the need for better implementation tools that support patient engagement and identified the necessity of optimizing the design of the self-management resources included in or with guidelines [112]. Thus, a variety of theories offer insight into how patients’ perceptions influence their behavior and can be used to design and then evaluate self-management guideline tools.

In all the existing models, we found no framework that contained all the dimensions [40–44]. The HTA framework was not specifically related to the TR intervention, but it allowed us to position the review at a broader level of assessment. If we had focused only on the intervention (the domain alone being restrictive), we would have excluded the other dimensions. We therefore took a broader perspective of the assessment by focusing on encompassing more domains rather than on the intervention.

For our secondary objective, we aimed to identify the phases of TR interventions in which assessment occurs. Our results showed a marked interaction between assessment domains and the distinct development phases of TR. This allowed us to highlight which domain was assessed at which phase, suggesting that it is inappropriate to assess all domains in a single phase. This TR assessment process can capture comprehensive, dynamic and complex evidence, crossing the various domains of assessment with the development phases of TR. While many TR assessments are still quite disconnected from each other and therefore fail to create a synergic effect in TR research efforts, one suggestion for interpretation would be that there is confusion or misunderstanding on the part of different stakeholders of the various assessment possibilities to be performed in the early stages of TR development.

Furthermore, through comparison with existing literature, we observed that this TR assessment process is comparable to the e-health evaluation model of Enam et al. (2018) [30]. For example, when e-health intervention is initiated at the *design* phase, the decisions are made solely based on the assessment of the *technological* and *cost* domains of technology development, whereas in this scoping meta-review, they also include *social aspects, organizational aspects*, *legal aspects* and *ethical analysis.* Therefore, our process proposes an additional specification not present in the evaluation model of e-health interventions in general.

This TR assessment process could become cumbersome because of high resource consumption, but it is not a prescription, just a way to show the progression of evidence in TR applications in a reliable manner. In that respect, the true value of this review is the fact that it suggests that more work is needed to make sure that other relevant domains in each phase of development are incorporated over time. As such the HTA model may be useful in identifying what domains may be necessary to strengthen as they are not currently sufficiently evaluated. In addition, since the HTA model did not integrate interventional aspects, a combination of two frameworks could have been a solution. From a multidisciplinary point of view, one could also address the mediators, moderators and mechanisms of change, in order to replicate one's findings [113].

Finally, only two reviews actually use the word *telerehabilitation*; thus, the field may be divided in terms of terminology. The impact of this term usage in relation to identifying a common evaluation agenda is a most relevant issue, again relating to the maturity of the field [114]. TR is intended to be multidisciplinary, and thus to serve different professions, which partly contributes to a division of knowledge at present. There is now a need for harmonization to understand the heterogeneity of TR. Each society has its own definition of the types of rehabilitation [115], and it might be relevant to consider a federation or a transversal definition that encompasses the multiple disciplines. At present, one possible interpretation would be that it is either a problem of maturity of the discipline or an inadequacy of a global and specific definition for each type of pathology (example: rehabilitation in musculoskeletal diseases is not the same as diabetes or obesity).

### Limitations

The main strengths of this review are the use of the scoping review methodology, which enabled coverage of a very broad range of topics; the comprehensive search strategy developed; and the rigorous quality assessment of each review by two independent researchers. However, there are a number of limitations that must be highlighted. First, a scoping meta-review can only report on literature that has been included in published reviews. Thus, some recently published primary research might not be included. Another limitation was that our electronic database searches may have missed relevant citations. This limitation is potentially due (1) to restriction of the search to English and French language publications and (2) to certain documents that may have been omitted, unknowingly and unintentionally. However, we have included and analyzed many journals in this scoping meta-review. Additionally, these TR reviews included only the five major groups of known chronic diseases that represent the highest rate of premature mortality, limiting the generalizability of the results. It would be interesting to determine whether this TR assessment process could be extended and applied to other chronic pathologies that require TR, such as osteoarthritis or stroke [116, 117].

Finally, this scoping meta-review shows that semantically, the remote delivery of rehabilitation is not homogeneous: the terms used include "m-health", "e-health", "telehealth", "web-based intervention/rehabilitation", "digital health intervention", "telemedicine" and "telerehabilitation". There is a use of multiple definitions and an apparent lack of solidarity in defining TR. How, then, do we – collectively – define TR? The confusion extends to other aspects of the TR domain [114]. According to Scott et al. (2013), it seems important to resolve the semantic issues around “e-health strategies” and identify barriers to TR, such as profession-centric nomenclature. Further discussion can then be pursued to ensure that the diversity of TR is understood and that the appropriate mix of specific solutions is brought to bear in response to defined health needs.

## Conclusions

This scoping meta-review reported on a large number of reviews that focused on assessing TR intervention for chronic diseases. By proposing and using a comprehensive assessment framework for TR, our results highlighted ten assessment domains and a list of the main related aspects. The different domains mobilized for assessment are not all studied with the same degree of interest. Furthermore, we showed that each of these assessment domains could appear at different phases of TR development, whereas current research generally focuses on one or a few assessment dimensions. These main contributions allow us to enrich this literature on the assessment of TR and propose new cross-disciplinary and complete method for the assessment of TR interventions.

Due to the challenge of integrating TR into the management of patients with chronic diseases, this framework could guide future studies in developing a research agenda on TR assessment with the aim of obtaining a comprehensive view of the assessment of TR. Thus, improved validation of evaluation methods could facilitate the transferability of results among similar studies and bring together the best evidence to assess TR interventions across a broad range of domains.

## Supplementary Information

**Additional file 1.**

**Additional file 2.**

**Additional file 3.**

**Additional file 4.**

**Additional file 5.**

## Data Availability

All data generated or analyzed during this study are included in this published article and its supplementary information files.

## Abbreviations

AMSTAR: Assessing the Methodological Quality of Systematic Review
CADTH: Canadian Agency for Drugs and Technologies in Health
HTA: Healthcare Technology Assessment
ICT: Information and communication technologies
NCD: Noncommunication diseases
PRISMA-ScR: Preferred Reporting Items for Systematic reviews and Meta-Analysis Extension for Scoping Review
RCT: Randomizes Controlled Trials
WHO: World Health Organization
