# Supplementary material for: Standardization of the assessment process within telerehabilitation in chronic diseases: a scoping meta-review
Source: BMC Health Serv Res. 2022 Aug 2;22:984. doi: 10.1186/s12913-022-08370-y (PMC9344755; doi:10.1186/s12913-022-08370-y)
Supplement: Supplementary file 1 — Additional file 1. [file 12913_2022_8370_MOESM1_ESM.pdf]

Additional File 1 Search Strategy as used in Web of Science on October 2, 2019

Search category	Search keywords
Telerehabilitation	TI=(telerehabilitation* OR tele-rehabilitation* OR "tele rehabilitation*" OR "remote rehabiliatation*" OR "virtual NEAR/4 rehabilitation*" OR "web-based NEAR/3 rehabilitation*" OR ehealth OR e-health OR mhealth OR m-health OR "app-based rehabilitation" OR telecare OR telecommunication OR telemonitoring OR "web-based NEAR/3 intervention" OR mobile)
Evaluation	TI=(evaluat* OR assess* OR apprais* OR effectiveness OR feasibilit*)
Chronic disease	TI=("chronic disease*" OR "noncommunicable disease*" OR "chronic illness*" OR "chronic respiratory disease" OR COPD OR "Chronic Obstructive Pulmonary Disease" OR Asthma OR Diabetes OR "Cardiovascular disease" OR hypertension OR "Pulmonary Heart Disease" OR cancer OR obesity)
Combination	#3 AND #2 AND #1

The steps followed: **1/** Telerehabilitation OR telemedicine OR … OR … (category 1); **2/** evaluation OR assess* OR… (category 2); **3/** chronic disease OR non-communicable disease OR chronic illness OR… (category 3); **4/** ti(category 1) AND ab(category 1); **5/** ti(category 2) AND ab(category 2); **6/** ti(category 3) AND ab(category 3); **7/** 4/ AND 5/ AND 6/
