# Supplementary material for: Standardization of the assessment process within telerehabilitation in chronic diseases: a scoping meta-review
Source: BMC Health Serv Res. 2022 Aug 2;22:984. doi: 10.1186/s12913-022-08370-y (PMC9344755; doi:10.1186/s12913-022-08370-y)
Supplement: Supplementary file 2 — Additional file 2. [file 12913_2022_8370_MOESM2_ESM.pdf]

Additional File 2 Main domains of assessment from the HTA Core Model (Lampe et al., 2008)

Domain of assessment	Description
Health problem and current use of technology	*"Deal with the health problems for which the technology under assessment is intended to be used (target conditions, target groups) and with the availability and patterns of use of the technology in question",* p. 37
Description and technical characteristics of technology	Should enable the user *"to quickly assess the relevance or applicability of the report for his own purposes. This includes the rationale behind the use of the technology and potential utility for patients",* p. 54.
Safety	Refers to *"side-effects, adverse events or adverse effects, complications, harms, risks and hazards, safety, tolerability and toxicity",* p. 61
Accuracy	*"Describes the potential of the technology to correctly distinguish those with the target condition from those without (diseased from non-diseased), or to reduce the uncertainty about the presence or absence of the target condition in subjects investigated",* p. 71
Clinical effectiveness	Specifies *"the improved accuracy of the diagnostic pathway where the technology is used; the impact of the use of the technology on therapeutic decisions; and the effectiveness of the therapies selected on the basis of the use of the technology",* p.84
Costs and economic evaluation	*"To inform value for money judgements about an intervention", "to provide information to improve decision-making in the health care sector with respect to priority-setting between different health technologies, both emerging, new and existing ones",* p. 91
Ethical analysis	Considers *"Prevalent morals, values and behavioral models of the society relevant for health technology assessment",* p. 101
Organizational aspects	*Focuses "on the delivery modes of technologies", "to find ways of influencing the behavior of a diffuse (disparate) group of managers and health professionals",* p. 128
Social aspects	"*Take the patient as a point of departure in its analysis of the manifold social implications of health technology. The focus is on the diverse social arenas where the patient lives and acts during the period of sickness and treatment",* p. 144
Legal aspects	*"Helps to focus the scrutiny to relevant legal sources, which may vary according to the nature of the technology in question",* p. 158
