# Supplementary figures and images for: Standardization of the assessment process within telerehabilitation in chronic diseases: a scoping meta-review
Source: BMC Health Serv Res. 2022 Aug 2;22:984. doi: 10.1186/s12913-022-08370-y (PMC9344755; doi:10.1186/s12913-022-08370-y)

**Additional file 3** Types of associated technological tools

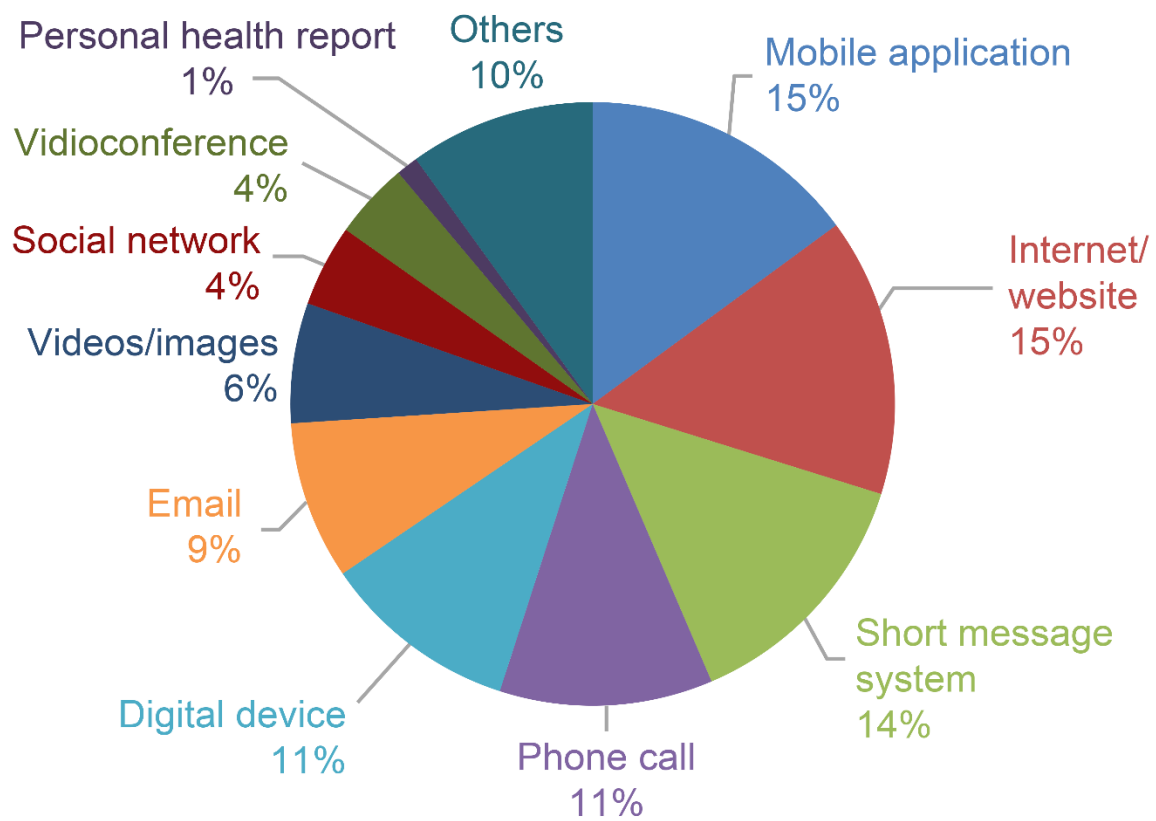

Supplement: Supplementary file 3 — Additional file 3. [file 12913_2022_8370_MOESM3_ESM.pdf]
