# Supplementary material for: Standardization of the assessment process within telerehabilitation in chronic diseases: a scoping meta-review
Source: BMC Health Serv Res. 2022 Aug 2;22:984. doi: 10.1186/s12913-022-08370-y (PMC9344755; doi:10.1186/s12913-022-08370-y)
Supplement: Supplementary file 4 — Additional file 4. [file 12913_2022_8370_MOESM4_ESM.pdf]

Additional file 4 Results of individual sources of evidence

Author	Design of review	Pathologies*	Aims	End-users	Domains of evaluation HTA**									
					**A**	**B**	**C**	**D**	**E**	**F**	**G**	**H**	**I**	**J**
Alessa et al., 2018	Qualitative syst. review	CVC	To assess the effectiveness of apps in lowering blood pressure as well as their usability and patients' satisfaction with their use.	Patient & health professional		x		x						
Arambepola et al., 2016	QSR and meta-analysis	D	To examine the effectiveness of interventions to change lifestyle behavior delivered via automated brief messaging	Patient	x	x		x					x	
Arnhold et al., 2014	Qualitative syst. review	D	To consider the number of newly released diabetes apps, range of functions, target user groups, languages, acquisition costs, user ratings, available interfaces, and the connection between acquisition costs and user ratings on all currently available diabetes apps for the iOS and Android operating systems	Patient & health professional	x	x	x	x						
Banbury et al., 2018	Qualitative syst. review	CVC; C; CRD; D; O	To review the literature to determine the feasibility, acceptability, effectiveness, and implementation of health professional-led group videoconferencing to provide education or social support or both in the home setting	Patient & health professional	x	x		x		x			x	
Bardus et al., 2015	Scoping review	O	To provide an up-to-date, comprehensive map of the literature discussing the use of mobile phone and Web 2.0 apps for influencing behavior related to weight	Patient	x	x								

			management (i.e., diet, physical activity [PA], weight control)										
Bassi et al., 2014	Qualitative syst. review	CVC; D; O	To investigate optimal methods for achieving lifestyle change in metabolic syndrome through a systematic literature review based on the Preferred Reporting Items for Systematic Reviews and Meta-Analyses (PRISMA) framework	Patient	x			x	x				x
Beatty et al.	Qualitative syst. review	CVC	To examine the existing literature on the use of mobile technology for cardiac rehabilitation and propose a framework for developing and evaluating mobile applications for cardiac rehabilitation	Patient & health professional									x
Beishuizen et al., 2016	QSR and meta-analysis	CVC; D; O	To evaluate whether Web-based interventions for cardiovascular risk factor management reduce the risk of cardiovascular disease in older people	Patient & health professional	x			x					x
Bellei et al., 2018	Qualitative syst. review	D	To identify and analyze studies regarding the diversity of applications designed for DM monitoring and treatment	Patient & health professional		x		x					
Bennett et al., 2014	Qualitative syst. review	O	To evaluate the efficacy of eHealth weight management interventions among overweight and obese racial/ethnic minority adults	Patient & health professional & caregiver	x								x
Beratarrechea et al., 2014	Qualitative syst. review	CRD; D	To discuss the impact of mobile health (m-health) on chronic disease outcomes (including morbidity, mortality, hospitalization rates, behavioral or lifestyle changes, process of care improvements, clinical outcomes, costs,		x		x	x	x				x

			patient-provider satisfaction, compliance, and health-related quality of life (HRQoL)) in low- and middle-income countries (LMIC)										
Birkhoff et al., 2016	Qualitative syst. review	CVC; CRD; D	To present a synthesis of the current qualitative research addressing the motivating factors, usability, and experiences of mobile health tracking applications (apps)	Patient		x		x					
Borries et al., 2019	Scoping review	D	To identify peer-reviewed medical literature on the use of telemedicine, assess its impact on self-management processes and the clinical outcomes of care, and delineate research gaps	Patient	x			x	x				x
Bossen et al., 2014	Qualitative syst. review	CVC; D; O	To summarize the evidence on the effectiveness of web-based PA interventions	Patient	x			x					
Brunton et al., 2015	QSR and meta-syntheses (issue meta-ethnography)	CRD	To systematically review and synthesize the findings from qualitative studies that investigated user perspectives and experiences of telehealth in COPD management in order to identify factors that may impact uptake	Patient & health professional	x	x		x					
Can Hou et al., 2016	QSR and meta-analysis	D	To investigate the effect of mobile phone applications (apps) on glycemic control (HbA1c) in diabetes self-management	Patient & health professional	x			x					x
Cassimatis et al., 2012	Qualitative syst. review	D	To review the effect of behavioral telehealth interventions on glycemic control and diabetes self-management	Patient & health professional		x		x					x

Chomutare et al., 2011	Qualitative syst. review	D	To study the salient features of mobile applications for diabetes care, in contrast to clinical guideline recommendations	Patient	x	x	x	x					
Clark et al., 2013	Qualitative syst. review	CVC	To identify and critique evidence for the effectiveness of alternative models of cardiac rehabilitation	Patient & health professional	x		x	x	x	x			x
Cotter et al., 2014	Qualitative syst. review	D	To identify studies that used Internet-based interventions to promote lifestyle modification	Patient & health professional	x	x		x		x			x
Cui et al., 2016	QSR and meta-analysis	D	To conduct a systematic review and meta-analysis of randomized controlled trials (RCTs) assessing the effect of mHealth apps on changes in hemoglobin A1c (HbA1c), blood glucose, blood pressure, serum lipids, and body weight in type 2 diabetes	Patient & health professional				x					x
Dale et al., 2016	Qualitative syst. review	CVC	To determine the effect of mHealth interventions on behavioral lifestyle changes and medication adherence for cardiovascular disease self-management	Patient & health professional	x		x	x					x
Dounavi et al., 2019	Qualitative syst. review	MC; D; O	To identify existing evidence on the efficacy of mobile health technology in facilitating weight management behavior, such as healthy food consumption and physical activity	Patient & health professional		x		x					x
Duff et al., 2017	Qualitative syst. review	CVC	To assess the application of BCTs in eHealth interventions designed to increase PA	Patient & health professional				x					

Fu et al., 2017	Qualitative syst. review	D	To assess the usability and clinical effectiveness of diabetes mobile applications (diabetes apps)	Patient & health professional		x		x			x		x
Gaikwad et al., 2009	Qualitative syst. review	CVC; C; CRD; D; O	To evaluate the feasibility and benefits of home-based information and communications technology-enabled interventions for chronic disease management, with emphasis on their impact on health outcomes and costs	Patient & health professional	x	x	x	x	x				
Gaveikaite et al., 2019	Umbrella	CRD	To examine the provision and efficacy of telehealth for COPD management to guide future decision-making	Patient & health professional	x		x	x	x				x
Goode et al., 2015	Qualitative syst. review	C	To evaluate the efficacy of physical activity, dietary, and/or weight control interventions in which telephone, short-message service, print, and/or the internet is the primary method of delivery	Patient & health professional	x	x	x	x		x			x
Gorst et al., 2014	Qualitative syst. review	CVC; MCR	To assess levels of uptake of home telehealth and the factors that determine whether patients do or do not accept and continue to use telehealth	Patient & health professional	x			x		x			
Haberlin et al., 2018	Qualitative syst. review	C	To explore the effects of eHealth in the promotion of PA	Patient	x	x		x					
Hailey et al., 2011	Qualitative syst. review	CVC; C; CRD	To systematically review the evidence on the effectiveness of telerehabilitation applications	Patient	x	x							x
Hall et al., 2015	Umbrella	CVC; CRD; D; O	To identify mobile text-messaging interventions designed for health	Patient	x			x					x

			improvement and behavior change and to derive recommendations for practice										
Hamilton et al., 2018	Qualitative syst. review	CVC	To assess the evidence on mHealth interventions for cardiac rehabilitation and heart failure management with respect to service and patient outcomes and cost effectiveness with a view to how mHealth could be utilized for rural, remote and Indigenous cardiac patients	Patient & health professional	x		x	x					x
Hamine et al., 2015	Qualitative syst. review	CVC; CRD; D	To evaluate the effectiveness of mHealth in supporting the adherence of patients to chronic disease management ('mAdherence') and the usability, feasibility, and acceptability of mAdherence tools and platforms	Patient & health professional	x	x		x	x				x
Hernandez et al., 2019	Qualitative syst. review	C	To assess the effectiveness of mHealth applications (apps) for self-management in improving pain, psychological distress, fatigue, or sleep outcomes	Patient	x			x					
Holtz et al., 2012	Qualitative syst. review	D	To understand the most common uses and functions of mobile phones in monitoring and managing diabetes, their potential role in a clinical setting, and the current state of research in this area	Patient	x	x		x					x
Huang et al., 2015		CVC	To determine the effectiveness of a telehealth-delivered cardiac rehabilitation (CR) intervention compared with center-based supervised CR	Patient & health professional	x								x

Hutchesson et al., 2015	QSR and meta-analysis	O	To evaluate the effectiveness of eHealth interventions for the prevention and treatment of overweight and obesity in adults.	Patient & health professional	x								x
Iribarren et al., 2017	QSR and meta-analysis	CVC; C; CRD; D; O	The body of evidence related to economic evaluations of mHealth interventions is assessed and summarized	Patient & health professional	x	x	x						
Isaacs et al., 2016	Qualitative syst. review	D	To examine (1) the research reporting practices and prevalence of ethnic minority patients included in telehealth RCTs and (2) the proportion of included RCTs that report using English language proficiency as a patient screening criterion and how and why they do so	Patient		x				x			
James et al., 2017	Qualitative syst. review	CVC; C; CRD; D; O	To present a systematic review of the participation of African Americans in e-Health/m-Health interventions, the diseases/health conditions targeted, and the recruitment and retention strategies used	Patient & health professional	x	x				x			
Joe et al., 2013	Qualitative syst. review	D	To report on the results of a review concerning the use of mobile phones for health (feasibility, acceptability, and effectiveness)	Patient & health professional	x			x					x
Joiner et al., 2017	Qualitative syst. review	D	To describe Diabetes Prevention Program (DPP)-based lifestyle interventions delivered via electronic, mobile, and certain telehealth (eHealth)	Patient & health professional	x	x				x			x

			means and estimate the magnitude of the effect on weight loss											
Jones et al., 2014	QSR and meta-analysis	CRD; D	To evaluate the current evidence on the use of mobile phones and SMS to deliver self-management interventions	Patient & health professional	x	x	x	x	x	x	x	x	x	x
Karoliina et al., 2010	Umbrella	O	To review the literature on tailored health communication and to present an interdisciplinary analysis of studies on second-generation tailored interventions aimed at behavior change in nutrition, PA, or weight management	Patient & health professional	x			x	x	x				
Kebede et al., 2017	Qualitative syst. review	D	To identify the active ingredients in electronic health (eHealth) interventions targeting patients with poorly controlled type 2 diabetes mellitus (T2DM) and the relevant outcomes	Patient	x			x					x	
Kitsiou et al., 2017	Scoping review	D	To critically appraise and consolidate evidence from multiple systematic reviews on the effectiveness of mHealth interventions to inform policy makers, practitioners, and researchers	Patient & health professional	x								x	
Kuijpers et al., 2013	Umbrella (meta review)	CVC; C; CRD; D	To systematically review the literature regarding interactive Web-based interventions, focusing on interventions aimed at increasing patient empowerment and PA	Patient & health professional	x	x		x						
Lee et al., 2018	Qualitative syst. review	CVC; C; CRD	To examine the effect of mHealth interventions on process measures as well as health outcomes in RCTs to improve chronic disease management	Patient & health professional		x		x	x				x	

Liang et al., 2011	Qualitative syst. review	D	To assess the effect of mobile phone intervention on glycemic control	Patient & health professional	x	x							x
Lundell et al., 2015	Meta-analysis	CRD	To investigate the effects of telehealthcare on PA level, physical capacity and dyspnea and to describe the interventions used		x			x					x
Ma et al., 2019	QSR and meta-analysis	CVC	To identify the delivery mode and strategies used by current eHealth interventions and examine their effect on blood pressure control, self-care behavioral outcomes and psychosocial well-being	Patient & health professional	x			x					x
Maramba et al., 2019	QSR and meta-analysis	C; D	To identify, explore, and summarize the current methods used in the usability testing of eHealth applications	Patient & health professional									
McKay et al., 2018	Scoping review	CVC; C; CRD; D; O	To investigate approaches to the evaluation of health apps to identify any current best practices	Patient & health professional									
Müller et al., 2016	Qualitative syst. review	CVC; D; O	To investigate the effectiveness of eHealth and mHealth interventions to promote PA and healthy diets in developing countries	Patient				x					
Neubeck et al., 2009	Qualitative syst. review	CVC	To determine the effectiveness of telehealth for coronary heart disease management	Patient & health professional				x					x
Nussbaum et al., 2019	QSR and meta-analysis	CVC; C; CRD	To conduct a systematic review to better define how medical mobile applications (apps) have been used in environments	Patient & health professional		x		x	x	x			x

			relevant to physical medicine and rehabilitation							
Palmer et al., 2018	Qualitative syst. review	CVC; C; CRD; D; O	To assess the effectiveness of smoking cessation, PA, diet, and alcohol reduction interventions delivered by mobile technology to prevent noncommunicable diseases (NCDs)	Patient & health professional		x	x		x	
Puigdomenech et al., 2019	QSR and meta-analysis	O	To identify the efficacy, safety, and effectiveness criteria used to assess weight control, overweight, and obesity management in mobile health (mHealth) interventions	Patient & health professional			x		x	x
Qudah et al., 2019	Qualitative syst. review	CVC; C; CRD; D	To explore the influence of mobile health applications on various dimensions of patient and healthcare provider relationships	Patient & health professional	x		x	x		
Raaijmakers et al., 2015	Qualitative syst. review	O	To provide insight into the effectiveness of technology-based interventions for weight loss and quality of life compared to standard care	Patient & health professional			x		x	
Ramadas et al., 2011	Qualitative syst. review	D	To provide a descriptive discussion of web-based behavioral interventions	Patient & health professional		x	x		x	
Ramsey et al., 2019	Qualitative syst. review	CRD	To review the content and quality of mHealth asthma management apps that are available to patients	Patient & health professional		x	x			
Rawstorn et al., 2016	Qualitative syst. review	CVC	To determine the benefits of telehealth exCardiac Rehabilitation (CR) on exercise capacity and other modifiable	Patient & health professional			x		x	

			cardiovascular risk factors compared with traditional exCR and usual care										
Rincon et al., 2017	QSR and meta-analysis	C	To review the scientific literature on mobile phone apps involving QoL and well-being (anxiety and depression symptoms) and analyze the clinical and technological characteristics, strengths, and weaknesses of these apps as well as patients' user experience with them	Patient & health professional				x				x	
Rodríguez et al., 2019		D	To assess the free mobile applications for the management of diabetes available in Spanish in the Spanish market	Patient		x	x	x					
Ruiz-Pérez et al., 2019	Qualitative syst. review	CVC	To examine the impact of interventions to improve cardiovascular disease healthcare provided to people living in rural areas	Patient & health professional		x		x	x				x
Seiler et al., 2017	Qualitative syst. review	C	To evaluate existing eHealth/mHealth interventions developed to help manage cancer-related fatigue (CRF) and summarize the best available evidence on their effectiveness	Patient & health professional	x			x					x
Stephens et al., 2013	Qualitative syst. review	CVC	To determine user satisfaction with and effectiveness of smartphone applications and text messaging interventions to promote weight reduction and PA	Patient & health professional				x					x
Sun et al., 2019		D	To examine the literature evaluating the use of mobile apps (stand-alone and text messaging/feedback) and to review the top-rated mobile apps' applicability	Patient & health professional		x	x	x					x

Tchero et al., 2019	Qualitative syst. review	D	To compare the effectiveness of telemedicine intervention with that of usual care	Patient & health professional	x		x						x
Triberti et al., 2019	QSR and meta-analysis	C	To explore the usage and effectiveness of eHealth resources to improve breast cancer patients'/survivors' quality of life and to describe if existing eHealth interventions addressed specific characteristics of breast cancer or employed a generic approach only	Patient & health professional				x					x
Unni et al.,	Qualitative syst. review	MCR	To summarize the use of technological aids and evaluate their impact on health outcomes (adherence and asthma control)	Patient & health professional				x					x
Vassilev et al., 2015	Qualitative syst. review	CVC; CRD; D	To identify the core mechanisms associated with successful telehealth implementation to consolidate the likely elements for ensuring a priori optimal design and deployment of telehealth interventions	Patient & health professional		x		x					
Wang et al., 2017		D; O	To examine published mHealth intervention studies for obesity and diabetes treatment and management to assess their effectiveness and provide recommendations for future research	Patient & health professional	x			x					x
Widmer et al., 2015	Meta-analysis	CVC	To assess the potential benefit of digital health interventions (DHIs) on outcomes (events, all-cause mortality, hospitalizations) and risk factors compared with non-DHIs	Patient & health professional									x

Winter et al., 2016	Qualitative syst. review	CVC	To examine the use of health behavior change techniques and theory in technology-enabled interventions targeting risk factors and indicators for cardiovascular disease (CVD) prevention and treatment	Patient & health professional		x		x						
Wu et al., 2018	Qualitative syst. review	D	To systematically review the literature and perform a meta-analysis comparing the clinical outcomes of telehealth and usual care in the management of diabetes	Patient & health professional		x								
Xiong et al., 2018	Qualitative syst. review	CVC	To systematically review existing evidence on the effectiveness of mobile health technology (mHealth) interventions in addressing medication adherence among people with hypertension	Patient & health professional	x			x					x	
Yasmin et al., 2016	Realist review	CVC; CRD; D	To carry out a systematic literature review on the impact of mobile health interventions - mobile phone texts and/or voice messages - in high, middle- and low-income countries to ascertain the impact on patients' adherence to medical advice as well as the impact on health outcomes	Patient & health professional				x					x	

*__Pathologies:__ CVC, cardiovascular disease; C, cancer; CRD, chronic respiratory diseases: D, diabetes; O, obesity
__**Domains of assessment from the EUnetHTA model:__ A, health issue and current use of technology; B, description and technical characteristics of the technology; C, cost and economic evaluation; D, social aspects; E, organizational aspects; F, legal aspects; G, ethical analysis; H, accuracy; I, clinical effectiveness; J, safety
