# Supplementary material for: Standardization of the assessment process within telerehabilitation in chronic diseases: a scoping meta-review
Source: BMC Health Serv Res. 2022 Aug 2;22:984. doi: 10.1186/s12913-022-08370-y (PMC9344755; doi:10.1186/s12913-022-08370-y)
Supplement: Supplementary file 5 — Additional file 5. [file 12913_2022_8370_MOESM5_ESM.pdf]

Additional File 5 A sample of the intervention characteristics (identified in 10 reviews by a check mark if they were present)

Author	Self-monitoring functionalities	Use of prompts/cues (reminders and alerts)	Educational information	Communication with others	Feedback	Stress management	Self-management (physical activity, diet, medication adherence, smoking)	Exercise training	Psycho-social support	Patient assessment	Others
Alessa et al., 2018	1	1	1	1	1	1	1				
Arambepola et al., 2016	1	1	1		1		1				
Arnhold et al., 2014	1	1	1	1	1		1				
Banbury et al., 2018			1	1				1	1		
Bardus et al., 2015	1	1	1	1	1		1				
Bassi et al., 2014	1		1		1						
Beatty et al.	1	1	1				1	1	1	1	
Beishuizen et al., 2016	1	1	1	1	1		1	1			Care management mobile coach support
Bellei et al., 2018	1	1	1	1	1	1	1				
Bennett et al., 2014	1		1	1	1	1	1	1	1		
